# Drug Price and Health Policy Knowledge Influence Prescription Behavior in Orphan Diseases: Pheochromocytoma and Paraganglioma As Prototypes of Orphan Drug Econometrics

**DOI:** 10.7759/cureus.80156

**Published:** 2025-03-06

**Authors:** Danny Q Le, Brittany N Burton, Christian J Tejeda, Joe C Hong, Jason S Lee, Nirav Kamdar

**Affiliations:** 1 Anesthesiology, University of California Los Angeles David Geffen School of Medicine, Los Angeles, USA; 2 Anesthesiology, Huntington Hospital, Pasadena, USA

**Keywords:** health policy and economics, paraganglioma, phenoxybenzamine, pheochromocytoma: rare disease, sodium nitroprusside

## Abstract

Introduction

Two federal drug policies, the Orphan Drug Act of 1983 (ODA) and the Unapproved Drug Initiative of 2006 (UDI), created market exclusivity and arbitrage opportunities in pharmaceutical pricing for two rare diseases, namely, pheochromocytoma and paraganglioma (PPGL). Historically, phenoxybenzamine and nitroprusside, amongst other medications, have been the first-line drugs for the perioperative and intraoperative management of patients undergoing PPGL resection. We aimed to assess the prescription behavior in perioperative pheochromocytoma management at an institution where providers have knowledge of orphan drug pricing.

Methods

We performed a retrospective cohort study of adult patients who underwent PPGL resection from January 2015 to September 2021. Patients in the study were seen at a tertiary academic medical center. 96 patients were included in this study and all patients underwent the standard University of California, Los Angeles (UCLA) perioperative surgical and anesthetic management. We assessed incidence of phenoxybenzamine and nitroprusside utilization as a perioperative blockade and as an intraoperative vasodilator, respectively. Statistical comparison of drug utilization was performed using the Cochran-Armitage test.

Results

Between 2015 and 2021, six (86%), 15 (75%), six (35%), seven (64%), two (17%), five (42%), and two (12%) patients received phenoxybenzamine as perioperative blockade, respectively. Similarly, between 2015 and 2021, one (14%), five (25%), 11 (65%), four (36%), 10 (83%), seven (58%), and 15 (88%) patients received alpha-1 selective blockade as perioperative treatment, respectively. The inverse change in phenoxybenzamine and alpha-1-selective agent utilization was statistically significant (p < 0.0001). Between 2015 and 2021, four (57%), two (10%), one (6%), zero (0%), zero (0%), zero (0%), and zero (0%) patients received nitroprusside as an intraoperative vasodilator, respectively. Similarly, between 2015 and 2021, three (43%), 18 (90%), 16 (94%), 11 (100%), 12 (100%), 12 (100%), and 17 (100%) patients did not receive nitroprusside as an intraoperative vasodilator, respectively. The decreasing nitroprusside utilization was statistically significant (p = 0.0004).

Conclusion

From 2015 to 2021, in the setting of the UDI and ODA, utilization trends of perioperative phenoxybenzamine and intraoperative nitroprusside decreased among the 96 patients undergoing PPGL resection. This study illustrates that provider knowledge of drug price increases can drive prescription behavior change. Provider knowledge of drug econometrics in rare diseases provides a platform for value creation in care delivery.

## Introduction

The Orphan Drug Act (ODA), first passed by the United States Congress in 1983, was created to stimulate drug development for rare diseases, or conditions affecting less than 200,000 people, in the United States [1,2]. The ODA incentivizes the development of treatments for small patient populations and has been heralded as increasing the number of rare disease treatments [1,2]. The law provides sponsors of approved orphan disease products with benefits including seven-year market exclusivity, a tax credit of 50 percent of the expenses of undertaking clinical experimentation, and research grants for the clinical testing of new treatments for orphan diseases [1-5]. Once a product has obtained orphan drug market exclusivity for the seven-year time period, the FDA cannot approve a generic drug application or new brand name for the same product and for the same rare disease indication [4,6,7].

As a result, pharmaceutical competition is limited by the exclusive marketing rights of the ODA, preventing other companies from marketing the same version of the drug unless clinical superiority can be proven [7,8]. Orphan drugs for rare diseases are now among the most expensive drugs on the market due to lower research and development costs, fast-track regulatory approval, and minimal competition even after ODA and patient market protection expire [5,7]. Additionally, orphan drugs can reach blockbuster status due to extension and multiplication of indications, effectively circumventing the orphan disease definition based upon size [7]. Wellman-Labadie and Zhou investigated issues associated with the ODA in 2010 and found that 9% of orphan drugs had already reached blockbuster status, with two-thirds having two or more indications [7]. The authors found that in 2006, orphan drugs with blockbuster status and one or more orphan designations brought in global sales of $58.7 billion [7]. Given the United States is the only major industrialized country which does not regulate prescription drug prices, these findings draw attention to the high treatment costs that fall on patients with orphan diseases.

Over two decades later, in 2006, Congress passed the Unapproved Drugs Initiative (UDI), a Food and Drug Administration (FDA) program aimed at reducing the number of drugs available on the market that lack FDA approval and could potentially be associated with safety and efficacy issues, ineffectiveness, or health fraud [9]. The law also sought to document supporting clinical data for several thousand drugs that remained available in the United States since before 1938 when the FDA began reviewing safety and effectiveness [9-12]. As an incentive, the FDA offered market exclusivity to the first manufacturer to receive approval of a previous unapproved drug [9-12].

In November 2020, the Department of Health and Human Services announced the end of the UDI citing the need to prevent drug manufacturers from enjoying artificial monopolies over older treatments and to protect patients from price increases and drug shortages [13]. Since the UDI allowed for a period of “de facto market exclusivity” following drug approval, manufacturers were afforded arbitrage opportunities to raise prices with little competition while not contributing substantial clinical trial data in most cases [14]. Gupta et al. found that once manufacturers of previously unapproved drugs received approval, they could market their product to payers and physicians as safer and more effective than unapproved competitor drugs, leading to competitor sales decreases and withdrawal from the market [9,14]. The authors discovered that the median average wholesale unit price for such drugs increased 37 percent for UDI drugs, and that 11 of 34 drugs studied increased in price by more than 128 percent [9,14].

The ODA and UDI policies have significantly increased drug prices for patients with the rare orphan diseases of pheochromocytoma and paraganglioma (PPGL), neuroendocrine tumors arising from adrenal medulla chromaffin cells or extra-adrenal neural crest progenitors, respectively [15,16]. During anesthesia and surgical resection of a PPGLs, the patient may experience severe hemodynamic instability including reduced cardiac output and associated systolic and diastolic dysfunction due to significant increases in plasma levels of norepinephrine and epinephrine [17-20]. Phenoxybenzamine, an irreversible non-specific alpha-adrenergic antagonist, was originally indicated by the FDA for malignant hypertension. Historically, an off-label indication for this drug was PPGL, but in 2015, phenoxybenzamine was resubmitted and accepted for treatment of PPGL.

Despite its prevalent use and greater blunting of hypertension, phenoxybenzamine is more expensive, associated with increased adverse effects in comparison with selective alpha blockers such as doxazosin, and does not translate to fewer cardiovascular complications [17-22]. For selective α-1 adrenergic blockers, the added advantages include ease to titrate the dose, lower drug cost, and lower incidence of reflex tachycardia, and postoperative hypotension [17,18]. Nitroprusside, a potent systemic vasodilator, is another first-line drug for management of PPGL during the perioperative and intraoperative periods [17,18,21-24]. Additionally, recent price increases for nitroprusside have increased consideration and potential physician behavior change toward the use of similar intravenous cardiovascular drugs with stable prices.

Health policy-driven financial consequences resulting from the combination of the ODA and UDI have contributed to the high prices of phenoxybenzamine and nitroprusside for patients with PPGL. Specifically, the UDI granted marketing exclusivity to manufacturers who demonstrate a new use for a drug or even a new dosing regime and if the new indication applies to an orphan disease under the ODA, the market exclusivity period can be seven years and lead to severe drug price increases. This topic is especially timely given the recent discontinuation of an FDA-approved modality, Azedra (iobenguane I-131 injection, solution) (Progenics Pharmaceuticals, North Billerica, USA), as well as the elevated prices of another effective agent, metyrosine [25, 26]. This study aims to evaluate prescription behavior change when providers understand the financial and policy consequences of these health policies for patients with PPGL. We hypothesized that provider knowledge of pharmaceutical orphan drug pricing would shift prescription and use behavior from first-line to second-line agents.

## Materials and methods

Data collection

We performed a retrospective cohort study of adult patients who underwent PPGL resection at the University of California, Los Angeles (UCLA) Department of Surgery, Division of Endocrine Surgery from January 1st, 2015 to September 30th, 2021. The Institutional Review Board (IRB) approval was obtained but given exempt status for the purposes of analyzing and retrospectively reporting our results (IRB#17-000048).

The effects of the ODA and UDI were explained to UCLA’s Anesthesiology and Perioperative Medicine and Endocrine Surgery Department during Grand Round Lectures on December 6th, 2017, with a goal of value-adding to patient care. All PPGL resections were performed at the Ronald Reagan UCLA Medical Center, Los Angeles, with standard UCLA perioperative surgical and anesthetic management. 

We collected the incidence of phenoxybenzamine or alpha-1 selective blocker (doxazosin, terazosin, prazosin) utilization as perioperative blockade and incidence of nitroprusside utilization as an intraoperative vasodilator. Other data collected include patient demographics: age, sex, and American Society of Anesthesiologists (ASA) physical status class.

Statistical analysis

Patient demographics were summarized between perioperative phenoxybenzamine and alpha-1 blocker cohorts; and intraoperative nitroprusside and no nitroprusside cohorts using mean (SD) or frequency (%) and formally compared using the independent samples t-test, Pearson’s chi-square, and Fischer’s exact test, as appropriate. Statistical comparison of perioperative blockade and intraoperative vasodilator drug utilization trend was performed using the Cochran-Armitage test. Data analysis was performed using JMP Pro v.15.0.0 statistical software (SAS Institute Inc., Cary, USA) and p-values <0.05 were considered statistically significant.

STROBE guidelines

Methods were conducted in accordance with the Strengthening the Reporting of Observational Studies in Epidemiology (STROBE) checklist for cohort studies.

## Results

A total of 96 patients underwent PPGL resection during the defined study period (Table 1).

**Table 1:** Comparison of demographics and ASA status between patients who received perioperative phenoxybenzamine and alpha-selective antagonists. Data presented as mean (SD) or n (%). *Statistically significant difference between groups. ASA: American Society of Anesthesiologists

	Phenoxybenzamine	Alpha-selective antagonists	P-Value
Total Patients, n	43	53	
Mean Age (SD), years	49.39 (17.07)	53.43 (17.73)	0.2602
Male, n (%)	20 (46.51%)	25 (47.17%)	0.9488
Female, n (%)	23 (53.49%)	28 (52.83%)	0.9488
ASA Class			0.3138
ASA Class 2, n (%)	5 (11.63%)	5 (9.43%)	
ASA Class 3, n (%)	37 (86.05%)	43 (81.13%)	
ASA Class 4, n (%)	1 (2.33%)	5 (9.43%)	

Perioperative blockade

**Figure 1:**
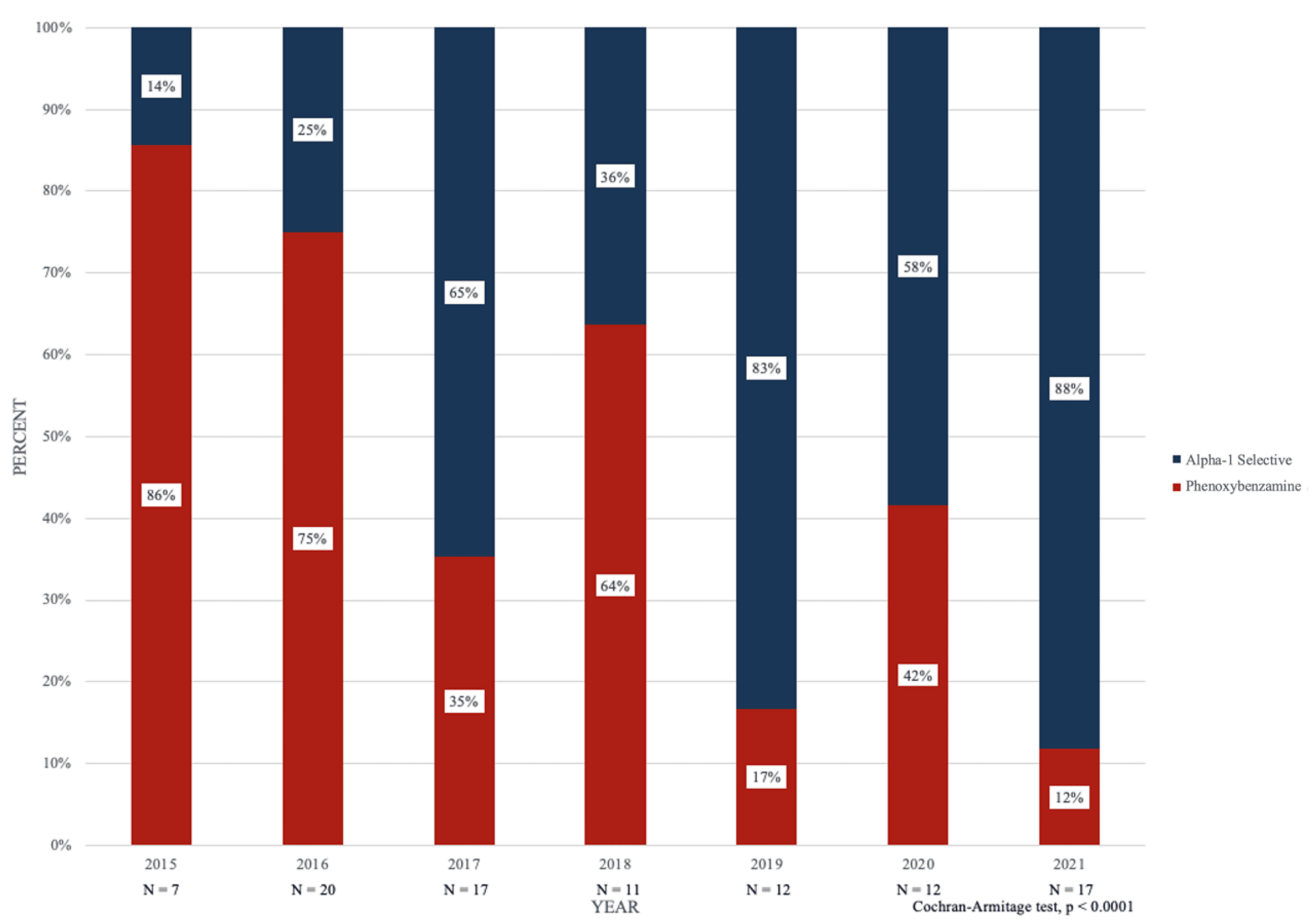
Phenoxybenzamine versus alpha-selective antagonists utilization incidence peri-pheochromocytoma-and-paraganglioma resection from 2015 - 2021.

A total of 43 (44.7%) patients received perioperative phenoxybenzamine and patient demographics including age, sex, and ASA physical status were found to be statistically similar (Table 1). Between 2015 and 2021, six (86%), 15 (75%), six (35%), seven (64%), two (17%), five (42%), and two (12%) patients received phenoxybenzamine as perioperative blockade, respectively. Similarly, between 2015 and 2021, one (14%), five (25%), 11 (65%), four (36%), 10 (83%), seven (58%), and 15 (88%) patients received alpha-1 selective blockade as perioperative treatment, respectively (Figure 1). The inverse change in phenoxybenzamine and alpha-1-selective agent utilization was statistically significant (p < 0.0001).

Intraoperative vasodilation

**Figure 2:**
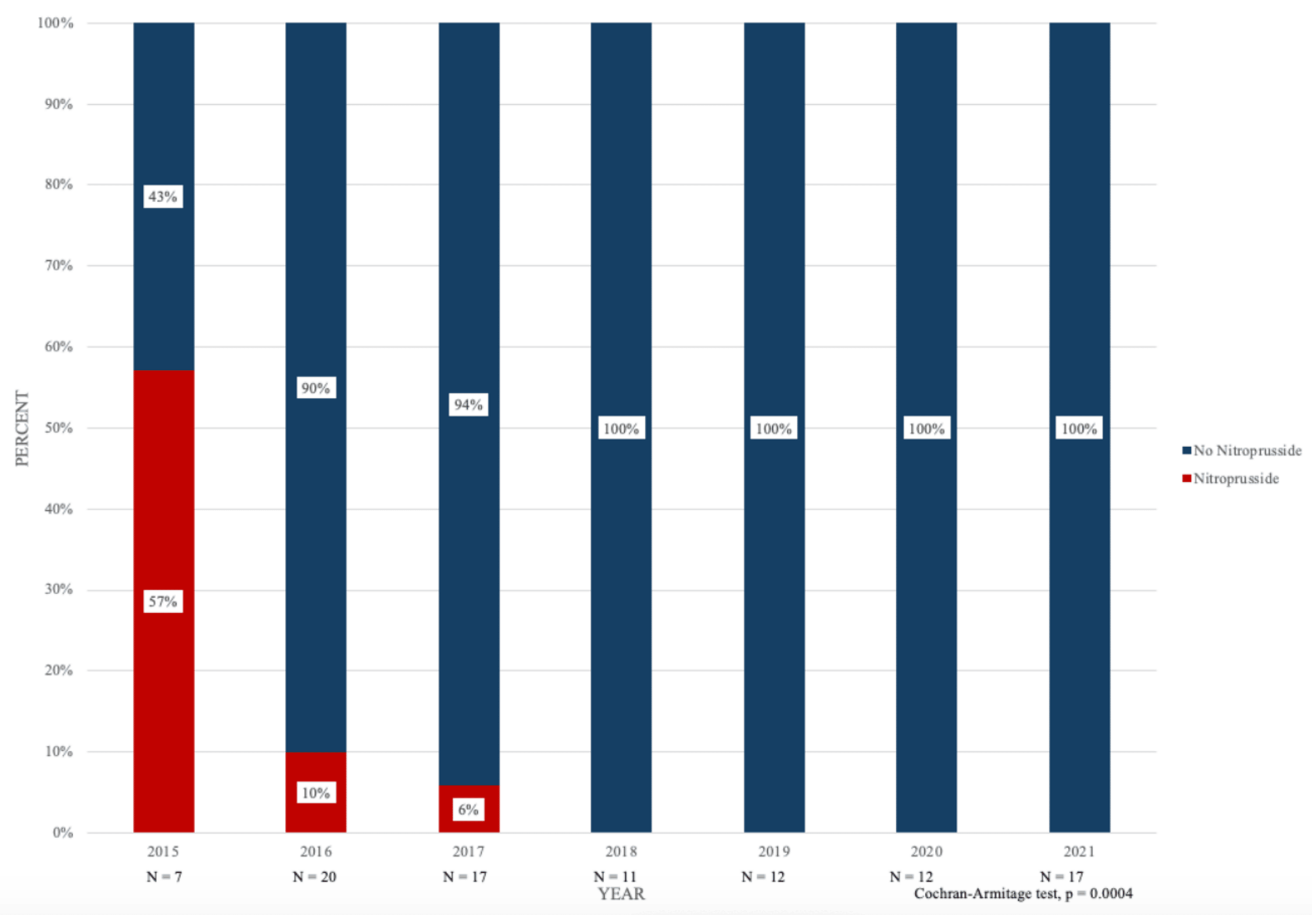
Intraoperative nitroprusside versus non-nitroprusside utilization incidence during pheochromocytoma and paraganglioma resection from 2015 - 2021.

A total of seven (7.2%) patients received intraoperative nitroprusside and patient demographics including age, sex, and ASA physical status were found to be statistically similar (Table 2). Between 2015 and 2021, four (57%), two (10%), one (6%), zero (0%), zero (0%), zero (0%), and zero (0%) patients received nitroprusside as an intraoperative vasodilator, respectively. Similarly, between 2015 and 2021, three (43%), 18 (90%), 16 (94%), 11 (100%), 12 (100%), 12 (100%), and 17 (100%) patients did not receive nitroprusside as an intraoperative vasodilator, respectively (Figure 2). The decreasing nitroprusside utilization was statistically significant (p = 0.0004).

**Table 2:** Comparison of demographics and ASA status between patients who received intraoperative nitroprusside and those who did not. Data presented as mean (SD) or n (%). *Statistically significant difference between groups. ASA: American Soceity of Anesthesiologists

	Nitroprusside	No Nitroprusside	P-Value
Total Patients, n	7	89	
Mean Age (SD), years	46 (7.59)	52.07 (17.96)	0.1030
Male, n (%)	3 (42.86%)	42 (47.19%)	0.8249
Female, n (%)	4 (57.14%)	47 (52.81%)	0.8249
ASA Class			0.6014
ASA Class 2, n (%)	1 (14.29%)	9 (10.11%)	
ASA Class 3, n (%)	5 (71.43%)	75 (84.27%)	
ASA Class 4, n (%)	1 (14.29%)	5 (5.62%)	

## Discussion

Our results for the two perioperative blockade groups show a statistically significant inverse change involving a marked decrease in phenoxybenzamine use and a sharp increase in alpha-1 selective agent blockade. Patient demographics are highly similar between the two perioperative blockade groups, which strengthens the data presented and eliminates potential confounding variables including age, sex, and ASA status. Between 2017 and 2018 and between 2019 and 2020, phenoxybenzamine use increased but drastically trended downward (86% to 13%) over the larger six-year time period studied. Conversely, between 2017 and 2018 and between 2019 and 2020, alpha-1 selective blockade decreased but significantly trended upward (14% to 88%) over the larger six-year time period studied.

For the two intraoperative vasodilation groups, our results showed a statistically significant decrease in nitroprusside utilization for the larger time period studied with decreases occurring between 2015 and 2018 until nitroprusside use bottomed out at 0% between 2018 and 2020. At UCLA, nitroprusside was also reorganized based upon price at the level of the pharmacy, which may have influenced physician behavior for using the drug to treat intraoperative hypertension secondary to PPGL. Patient demographics were also highly similar for the two intraoperative vasodilation groups, strengthening the results and reducing the possibility of confounding variables. The increase in non-nitroprusside drug use between 2015 and 2016 from 37.5% to 90% was particularly striking. It is important to highlight that nitroprusside prices increased by a factor of 30 per 50 mg from $27.46 in 2012 to $880.88 in 2015 [27].

The data from the study strengthens the argument that nitroprusside price increases have influenced provider nitroprusside utilization behavior. The decrease in nitroprusside use occurred even more rapidly and drastically than the decrease in phenoxybenzamine use for perioperative blockade. Our study highlights that physicians are actively integrating an understanding of drug econometrics with clinical judgment to provide affordable, high-quality value-add care to patients. With the goal of value-added care, PPGL leadership at UCLA delivered a series of grand rounds lectures and seminars explaining the effects of the ODA and UDI policies on drug prices. These were seminal and instigating events that encouraged physicians at our institution to change their prescription behaviors from first-line to second-line agents for the management of pheochromocytoma.

Our study also supports the hypothesis that the provider's understanding of the health policies impacting orphan drug costs influences their prescription behavior toward the utilization of safe and affordable second-line agents. It is important for healthcare providers to understand the current health policy landscape as it pertains to their patients’ care and access to affordable treatment options. Our findings may also refute the claim that price increases do not reduce patients’ access to medications historically utilized as first-line agents to manage their orphan diseases. The current findings also support pursuing opportunities to find preventative solutions to orphan drug price increases in the setting of federal policies.

Robust research studies should be undertaken to further understand the safety profiles and efficacies of second-line agents and other alternative treatments. There are current studies being conducted to compare hemodynamic instability, morbidity, mortality, cost, and quality of life between patients intraoperatively blocked with phenoxybenzamine versus second-line agents such as doxazosin for PPGL. Phenoxybenzamine, a significantly more expensive non-selective alpha blocker, is associated with increased adverse effects in comparison with selective alpha blockers such as doxazosin [17,18].

Although statistically significant results were achieved for both the perioperative blockade and intraoperative vasodilation groups, limitations of the study include a small sample size, which may limit the statistical power to detect smaller but clinically meaningful differences and trends in drug utilization.

Another limitation is that our study is limited to a single institution, which may reduce the generalizability of the findings to other healthcare settings with different prescribing behaviors, policies, and patient demographics. Including patients undergoing PPGL resection from other large healthcare centers across multiple populations would increase the power of the study and allow for greater generalization of results in future studies. Specifically, there may be differences in the orphan drug markets between states as well as drug utilization practices based on patient demographics and the health insurance landscape across different regions.

Additionally, no data prior to 2015 was analyzed to better understand previous trends in drug utilization behavior. Analyzing retrospective drug use data from prior to 2015, particularly for the nitroprusside versus no nitroprusside groups, would be helpful in understanding the origin of the decrease in nitroprusside use coinciding with the initiation of the price increases in 2012. Also, the retrospective cohort study design presents investigative flaws as well, such as selection bias and potential confounding variables that cannot be fully controlled, which could impact the validity of the observed associations.

The change in nitroprusside and phenoxybenzamine utilization is most likely associated with the changes in drug price, but we acknowledge tighter preoperative blood pressure management with alpha-selective agents may lead to less need for intraoperative vasodilators like nitroprusside. Additionally, other considerations, including better alternative drug agents or closer preoperative optimization, can be possible associated factors. Further studies examining these studies may provide greater insight.

Lastly, our study does not account for long-term patient outcomes associated with the changes in prescription behavior, such as postoperative hemodynamic stability or complication rates, which are crucial to assessing the clinical impact of the shift to second-line agents.

## Conclusions

From 2015-2021, in the setting of the UDI and ODA policies, utilization trends of perioperative phenoxybenzamine and intraoperative nitroprusside decreased among the 96 patients undergoing PPGL resection at UCLA. This study illustrates that provider knowledge of policy-driven increases in drug prices can impact drug-usage behavior for patient care toward value-based pharmaceutical options in the perioperative and intraoperative periods and provides a platform for value creation in care delivery. The pharmacoeconomic consequences of the UDI were evaluated, and its dissolution indicates a positive step toward affordable, patient-centered care for rare diseases. Future studies should continue to investigate the safety and efficacy of second-line agents for perioperative and intraoperative pheochromocytoma care as well as drug utilization trends in coming years.
